# Preventing hospital admissions by reviewing medication (PHARM) in primary care: design of the cluster randomised, controlled, multi-centre PHARM-study

**DOI:** 10.1186/1472-6963-11-4

**Published:** 2011-01-07

**Authors:** Anne J Leendertse, Fred HP de Koning, Alex N Goudswaard, Andries R Jonkhoff, Sander CA van den Bogert, Han J de Gier, Toine CG Egberts, Patricia MLA van den Bemt

**Affiliations:** 1 SIR Institute for Pharmacy Practice and Policy, Theda Mansholtstraat 5b 2331 JE, Leiden, the Netherlands; 2 Utrecht Institute for Pharmaceutical Sciences (UIPS), Division of Pharmacoepidemiology & Clinical Pharmacology, Faculty of Science, Utrecht University, PO Box 80 082, 3508 TB, Utrecht, the Netherlands; 3 Patient Safety Center, University Medical Center Utrecht, PO Box 85.500, 3508 GA, Utrecht, the Netherlands; 4 Kring Pharmacies, PO Box 210, 5201 AE, 's-Hertogenbosch, the Netherlands; 5 Dutch College of General Practioners (NHG), PO Box 3231, 3502 GE Utrecht, the Netherlands; 6 Jonkhoff huisartsenpraktijk, Joh. de Breukstraat 42, 2021 HB, Haarlem, the Netherlands; 7 Department of Pharmacotherapy and Pharmaceutical Care, University of Groningen, Antonius Deusinglaan 1, 9713 AV, Groningen, The Netherlands; 8 Department of Clinical Pharmacy, University Medical Center Utrecht, PO Box 85.500, 3508 GA, Utrecht, the Netherlands; 9 Department of Hospital Pharmacy, Erasmus Medical Center, PO Box 2040 3000 CA, Rotterdam, the Netherlands

## Abstract

**Background:**

Medication can be effective but can also be harmful and even cause hospital admissions. Medication review or pharmacotherapy review has often been proposed as a solution to prevent these admissions and to improve the effectiveness and safety of pharmacotherapy. However, most published randomised controlled trials on pharmacotherapy reviews showed no or little effect on morbidity and mortality. Therefore we designed the PHARM (Preventing Hospital Admissions by Reviewing Medication)-study with the objective to study the effect of the total pharmaceutical care process on medication related hospital admissions and on adverse drug events, survival and quality of life.

**Methods/Design:**

The PHARM-study is designed as a cluster randomised, controlled, multi-centre study in an integrated primary care setting. Patients with a high risk of a medication related hospital admission are included in the study with randomisation at GP (general practitioner) level. We aim to include 14200 patients, 7100 in each arm, from at least 142 pharmacy practices.

The intervention consists of a patient-centred, structured, pharmaceutical care process. This process consists of several steps, is continuous and occurrs over multiple encounters of patients and clinicians. The steps of this pharmaceutical care process are a pharmaceutical anamnesis, a review of the patient's pharmacotherapy, the formulation and execution of a pharmaceutical care plan combined with the monitoring and follow up evaluation of the care plan and pharmacotherapy. The patient's own pharmacist and GP carry out the intervention. The control group receives usual care.

The primary outcome of the study is the frequency of hospital admissions related to medication within the study period of 12 months of each patient. The secondary outcomes are survival, quality of life, adverse drug events and severe adverse drug events. The outcomes will be analysed by using mixed-effects Cox models.

**Discussion:**

The PHARM-study is one of the largest controlled trials to study the effectiveness of the total pharmaceutical care process. The study should therefore provide evidence as to whether such a pharmaceutical care process should be implemented in the primary care setting.

**Trial Registration:**

Trial number: NTR 2647

## Background

Drug regulatory authorities give a medicine marketing authorisation if the balance between efficacy and safety is considered positive, based on the available evidence in the studied population. On the level of the individual patient treated with one or more drugs in daily clinical practice, it is, however, hard to predict whether the positive effects outweigh the negative effects. Individual (patho)physiological and psychological characteristics of the patient, concomitantly used medication as well as the treatment setting, can largely modulate pharmacokinetics, pharmacodynamics and behaviour of the patient and thereby treatment outcomes. Unfortunately for some patients the net effect of pharmacotherapy is not positive but harmful. For example, several studies have shown that 3-5% of all hospital admissions is medication related[1].

The Dutch multi-centre HARM-study on medication-related hospital admissions, showed that almost half (46%) of these admissions were considered as potentially preventable admissions. Patients who were especially at risk of medication-related admissions were elderly patients with multiple drug use, who are cognitively impaired or who are non-adherent to their pharmacotherapy[1-5].

Medication review or pharmacotherapy review has often been proposed as a solution to improve the effectiveness and safety of pharmacotherapy[2]. However, most published randomised controlled trials on pharmacotherapy reviews showed no or little effect on morbidity and mortality[6,7]. These studies differed largely with respect to the nature and extensiveness of the review techniques, the outcomes studied, setting and follow-up time and results are therefore difficult to compare. One study reported an increase in emergency readmissions[8], while in the other studies there is no suggestion that patients were harmed by the interventions[9,10], and even some consistency in suggesting that falls[11] and hospital admissions[12,13] might be reduced. Potentially relevant elements in these studies were a review of a fully available medical and drug history, structured pharmaceutical care plan approach, combined effort of pharmacist and GP and involvement and commitment of the patient.

Hence there is still a need for large studies to evaluate the effectiveness of a so called patient centred pharmaceutical care process, including reviewing pharmacotherapy, formulating a pharmaceutical care plan and monitoring and follow up evaluating of pharmacotherapy, in daily practice of an integrated primary care setting. Therefore we designed the PHARM-study with the objective to study the effect of the total pharmaceutical care process on medication related hospital admissions and on adverse drug events, survival and quality of life in patients with a high risk of medication related hospital admissions. This study is a practice based, cluster randomised controlled intervention study in an integrated primary care setting in the Netherlands. The study design with its explanation and definition of the different steps in of the pharmaceutical care process will be described in this paper. The results of the study will be presented in a separate paper.

## Methods/Design

### Participants

Only patients at high risk for a medication-related hospital admission are included in the study. To identify these patients we used risk factors from the HARM study as inclusion criteria[2]. This resulted in four criteria regarding age, polypharmacy, type of drug class used and non-adherence[14]. (See table 1 for detailed information on inclusion criteria) Patients could be included if they met all four of these criteria

**Table 1:** Definition of the inclusion criteria of the PHARM-study

Criterion	Definition
65 years or older	65 Years of age or older, at the time of inclusion.

polypharmacy	Five or more drugs with different chemical substances. Every separate drug is prescribed and dispensed, at least 3 times in the 12 months before inclusion and dispensed at least once in the 6 months before inclusion.

ATC A or ATC B drug	One or more drugs from the Anatomical Therapeutic Chemical (ATC)[25] class A; "alimentary tract and metabolism" or ATC class B; "blood and blood forming organs". At least one of each drug prescribed and dispensed in the 12 months before inclusion.

non-adherence	At least one drug with a refill rate below 0.8 or above 1.2 This refill rate was calculated by dividing the number of dispensed daily doses, by the number of days between the first and the last prescription dispensed. The refill rate was calculated for all chronically prescribed drugs, as defined above under polypharmacy, of which at least 60 daily doses were dispensed in the 12 months before inclusion. The number of dispensed daily doses was calculated by summing the dispensed daily doses between the first to the last prescription date and multiplying it by factor 1.1 to correct for irregular drug use and early collection of the prescription. The refill rate was only calculated for oral and inhalation drugs with a clearly prescribed dose regimen. It was therefore not calculated for PRN (Pro Re Nata = as needed) drugs.

The number of included patients is limited to the capacity of the GP and the pharmacist. If too many patients are eligible for inclusion, random selection by computer based on patient serial number, is used within the eligible group of one GP and pharmacist, to comply with the limit. The pharmacist and GP both can include patients in 2008 and 2009, with a follow-up period of 12 months.

Patients are excluded if they are resident in a nursing home, if their life expectancy is less than three months or if they will not give informed consent. Patients have to be withdrawn from the study when they are no longer a patient of the participating GP or pharmacist. This is possible when a patient moves to another area or to a nursing home or when a patient withdraws his/her informed consent.

The study is conducted in agreement with the principles of the Declaration of Helsinki (Edinburgh 2000) and was approved by the medical ethical review board of METOPP (Medisch-Ethische Toetsing Onderzoek Patiënten en Proefpersonen). The patient's pharmacist explains the procedure, possible benefit and burden of participation in the study to each patient and provides an informed consent form approved by METOPP. The patient is asked to sign the form prior to inclusion into the study. Patient data from this study are coded by the patient's own pharmacist. Analysing and publication of the results of this study will be performed anonymously.

### Study setting

The study is carried out from 2008 to 2010 in an integrated primary care setting, by the patient's own GP, pharmacist and practice nurse if available. All Dutch GPs, practice nurses and pharmacists working in primary care will be informed about the study and invited to participate by mail, by websites, by several articles in pharmacy and GP journals and by several presentations at regional meetings or at national symposia. They are asked to cooperate as a group of at least one pharmacist and at least two GPs. This cooperation includes sharing data from electronic medical records required for the study. The GPs are allocated as an intervention GP or a control GP, by random selection.

### Support

Before and during the intervention period the GP and the pharmacist are offered support by training, education and a web based community of practice with a helpdesk on pharmacotherapy. The web-based community of practice offers a place of sharing information and experiences within the group of participants, provides all materials for the study and establishes for the researchers a place to encourage and support the participants and monitor the progress of the study. The pharmacist receives training on communication skills focussing on the pharmaceutical anamnesis, motivation of patients and communicating with the GP. The GP and the pharmacist participate together in at least three workshops on the study protocol, assessment of pharmacotherapy and the assessment of adverse drug events. The helpdesk is managed by a clinical pharmacist with support of experts on pharmacotherapy, medication safety and care of the elderly.

The GP from the control patients does not received any additional training or support, other than generally in usual care.

### Intervention

**Figure 1:**
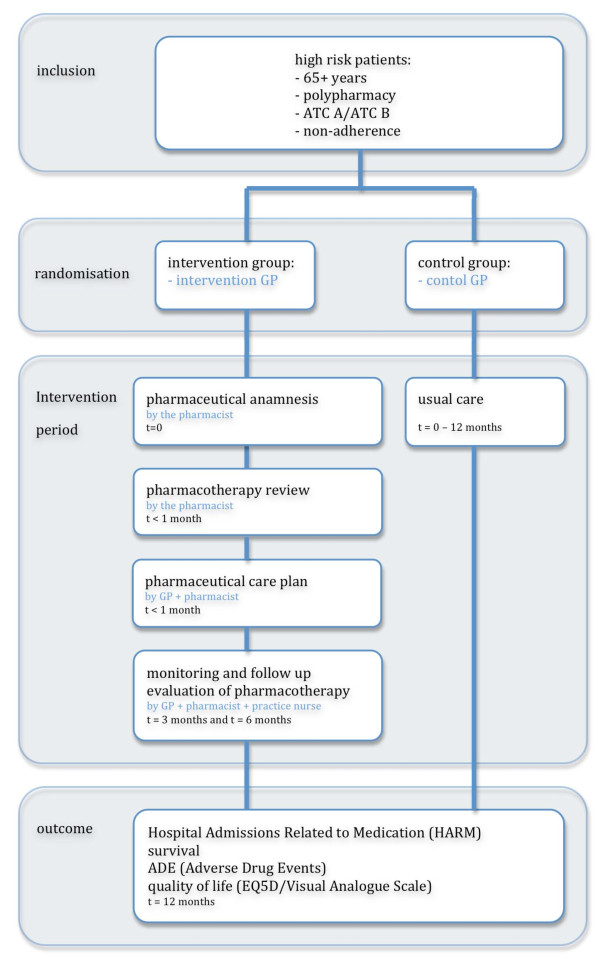
**Design of the PHARM-study**.

The intervention consists of a patient-centred, structured, pharmaceutical care process. This process consists of several steps, is continuous and occurs over multiple encounters of patients and clinicians. The steps of this pharmaceutical care process are a pharmaceutical anamnesis, a review of the patient's pharmacotherapy, the formulation and execution of a pharmaceutical care plan to combine with the monitoring and follow up evaluation of the care plan and pharmacotherapy (see figure 1).

#### Pharmaceutical anamnesis

The purpose of the pharmaceutical anamnesis is to gather information from the patient and its drug therapy. The anamnesis is performed by the pharmacist who meets the patient at home or in a private consultation room at the pharmacy or at the GP practice. During the pharmaceutical anamnesis the patient's medication use and experiences therewith are discussed, including the patient's current pharmacotherapy, drug history, drug use, possible allergies or intolerabilities and concomitant use of OTC drugs or other health products, but also the patient's beliefs, perceptions, understandings, attitudes, and concerns of the pharmacotherapy.

#### Pharmacotherapy review

The purpose of the pharmacotherapy review is to identify potential drug therapy related problems, and pharmaceutical care issues. A pharmaceutical care issue is defined as an element of a patient's drug related need, which can lead to a drug therapy related problem. We define a drug therapy related problem as any undesirable event or risk thereof, experienced by the patient that involves or is suspected to involve pharmacotherapy and that actually or potentially interferes with the desired patient outcome[15,16]. The pharmacist identifies these possible drug therapy related problems (DTPs) by combining sociological, pathophysiological and pharmacological knowledge of the patient in a systematic way. This structure is derived from the classification by Strand et al. as defined and described in table 2 and in table 3. The pharmaceutical care issues are described in table 4.

**Table 2:** Classification and definition of drug therapy problems

Category of drug therapy problem	Question to identify drug therapy problem	Drug therapy problem	Definition
Indication	Does the patient have an indication for each of his/her drug therapies, and is each of the patient's indications being treated with drug therapy?	Additional drug therapy required	The patient has a medical condition or is experiencing symptoms that requires the initiation of new or additional drug therapy or is at a high-risk to develop a new medical condition for which additional drug therapy is indicated.
		Unnecessary drug therapy	The patient is taking drug therapy that is unnecessary given his or her present condition or the patient is at risk to develop a new medical condition that is a result of taking an unnecessary drug for which there is no valid medical indication.

Effectiveness	Are the drug therapies effective for his/her medical condition? Are the intended outcomes of drug therapy reached?	Ineffective drug therapy	The drug product is not being effective at producing the desired response. The patient is not experiencing the intended positive outcome from a certain drug regimen or the intended outcome is not reached. Or an alternative drug therapy has a higher probability of producing the desired outcome, or an alternative drug therapy is equally effective but less expensive.
		Dosage too low	The patient has a medical condition for which too little of the correct drug is being taken to produce the desired beneficial outcome or the patient is at risk to develop a new medical condition because too little of the correct drug is being taken to expect a beneficial outcome. The patient's drug concentration in the body can be below the desired therapeutic range or the timing of prophylaxis can be inadequate for the patient or dose and interval can be inadequate for the patient or drug, dose, route or formulation conversions were inadequate for the patient.

Safety	Are the drug therapies as safe as possible? Is everything done to keep them as safe as possible?	Adverse drug event	The patient has a medical condition or is experiencing symptoms or is at risk of developing a medical condition which is undesired effect and is related to the drug therapy. This can be an idiosyncratic reaction to the drug, an allergic reaction to the drug or a pharmacologically expected reaction to the drug, possible due to a medication error.
		Dosage too high	The patient has a medical condition for which too much of the correct drug is being taken or the patient is at risk to develop a new medical condition because too much of the correct drug is being taken. The patient's drug concentration in the body can be above the desired therapeutic range or the drug dose escalating can be too rapidly or there can be drug accumulation from chronic administration or dose and interval can be inadequate for the patient or drug, dose, route or formulation conversions were inadequate for the patient.

Drug use	Is the patient able and willing to comply with the drug therapies as prescribed? Are the drug therapies as convenient as possible to the patient?	Drug use problem	Drug use problem is defined as the patient's inability or unwillingness to take a drug regimen that the GP, pharmacist or other health care provider has clinically judged to be appropriately indicated, adequately efficacious and able to produce the intended outcomes without any undesired effects.

**Table 3:** Description of drug therapy problems

Drug therapy problem	Common causes of drug therapy problem	Examples
Additional drug therapy required	• A medical condition requires the initiation of drug therapy. • Preventive drug therapy is required to reduce the risk of developing a new condition (according to the national guidelines). • A medical condition requires additional pharmacotherapy to produce an additive of synergistic effect.	• The patient is suffering from pain with no analgesic therapy. • A patient with chronic heart failure due to left ventricular systolic dysfunction, without an ACE-inhibitor or an angiotensin receptor blocker. • A patient with atrial fibrillation without antithrombotic therapy. • Calcium and vitamin D supplements for a patient with osteoporosis who is already taking a bisphosphonate.

Unnecessary drug therapy	• There is no current valid medical indication for the drug therapy for the individual patient. • Multiple drug products are being used for a medical condition that requires single drug therapy. • The medical condition is more appropriately treated with non-drug therapy or lifestyle changes. • Drug therapy is being taken to treat an avoidable adverse event associated with another medication. • Lifestile ( e.g. drug abuse, alcohol use, diet, smoking) is causing the problem.	• A patient is using a low dose of aspirin without a high risk of cardiovascular disease or any signs of a cardiovascular disease. • A patient is using three different laxative products in an attempt to treat his constipation. • A patient is using a benzodiazepine every night as a hypnotic drug for three years while it is better to recommend alternative sleeping patterns, sleep hygiene and exercise. • A patient is using furosemide to prevent swollen ankles. • A patient is using paracetamol combined with codeine (500/10) and is suffering from constipation which is treated with lactulose and bisacodyl occasionally. • A patient uses a protonpump inhibitor to treat dyspepsia associated with alcohol abuse.

Ineffective drug therapy	• The drug is not effective for the medical problem. • The drug product is not the most effective for the indication being treated. • The formulation of the drug products is inappropriate. • The drug is not effective because of the characteristics of the patient. (e.g. renal impairment, hepatic function)	• A patient is using an antibiotic for a common cold (viral infection). • A patient with benign prostatic hyperplasia uses doxasozine for more than four years. • • A patient with severe COPD uses salbutamol in a turbuhaler. • A patient with renal impairment uses a thiazide to lower the blood pressure.

Dosage too low	• The dose is too low to produce the desired outcome. • The dosage interval is too long to produce the desired outcome. • A drug-drug interaction reduces the amount of active drug available and the dose is not adjusted too produce the desired outcome. • The duration of the drug therapy is too short to produce the desired outcome.	• A patient is prescribed simvastatine 10 mg every other day after a myocardial infarction. • A patient uses 500 mg paracetamol, only twice a day, to control chronic pain in osteoarthritis. • A patient uses 375 mg amoxicilline, only once a day, to treat an airway infection. • A patient uses acenocoumarole and vitamin K. • A patient uses paroxetine for 4 days to treat anxiety.

Adverse drug event	• The drug causes an undesirable reaction that is not dose-related. • A safer product is required due to risk factors. • A drug interaction, with another drug or food, causes an undesirable reaction that is not dose-related. • The drug is contraindicated due to risk factors or other diseases. • The drug causes an allergic reaction. • A drug dosage was increased or decreased too fast. • A drug alters the patient's laboratory test results due to interference from a drug he/she uses.	• A patient on a low dose of aspirin is experiencing bruises. • An elderly patient uses flurazepam to sleep and is experiencing drowsiness at day time. • A patient who uses methotrexate gets prescribed co-trimoxazole for an infection (increase of anti-folate effect which can result in haematopoietic suppression). • A patient gets prescribed indometacin to control chronic pain, which is contraindicated because of his/her history with a peptic ulcer. • A patient is prescribed flucloxacillin for a dermal infection and develops a rash after the second dose. • A patient who uses prednisolone 20 mg every day for the last 6 months for arthritis symptoms iss instructed to take 10 mg for 2 more days and then discontinues the medication. • A patient had high blood glucose levels, due to the start of prednisolone therapy. • Positive ketone test in urine due to captopril use.

Dosage too high	• The drug causes an undesirable reaction due to too high dose. • The dosing frequency of the drug is too short. • The duration of drug therapy is too long. • The drug dose is too high in the patient because of its characteristics (excretion). • A drug-drug interaction occurs resulting in a toxic reaction to the drug. • The dose of the drug was administered too rapidly.	• A patient develops bradycardia resulting from a high (0.5 mg) daily dose of digoxine. • Hyperkaleamia after a dose of amiloride 10 mg three times a day. • A patient who experienes nasal congestion uses a nasal spray with xylometazoline for four weeks. • A patient with impaired renal function (CrCl: 20 ml/min) is prescribed a normal dose of 300 mg allopurinole a day, which causes nausea. • A patient has an increased INR after given metronidazole while also using acenocoumarole. • Cardiac arrest after infusion of a bolus of potassium phosphate (5 ml intravenously) instead of slow infusion.

Drug use problem	• • The patient does not understand the instructions. • The patient prefers not to take the medication. • • The patient forgets to take the medication. • • The patient cannot administer the drug appropriately him/herself. • • The drug therapy does not comply with the lifestyle of the patient. • • The patient has no access to the medication.	• The patient uses naproxen only when pain is unbearable while it is prescribed three times a day. • The patient is afraid of taking fluvoxamine because of possible side-effects. • The patient forgets to take his antihypertensive medication. • The patient is unable to administer the timolol eyedrops for her glaucoma. • A patient does not take furosemide because of attending activities at home. • A patient is not able to fetch the medication at the pharmacy.

**Table 4:** Classification and definition of pharmaceutical care issues

Pharmaceutical care issue	Definition
Monitoring	An intermittent (regular or irregular) series of observations in time, carried out to determine the effectiveness, safety and adherence of the drug therapy.

Drug-drug interaction	A combination of two or more drugs, administered by one patient that can result in a modification of the effect of at least one drug. The effect may be an undesired effect or a lack of effect of the drug.

Contra-indicated drug	A drug that is undesired because of the medical condition of the patient, which can lead to an adverse drug event or a lack of effect of the drug or a worsening of the medical condition of the patient.

Lifestyle	The lifestyle of the patient that could interfere with effective and safe drug therapy or that could result in non-adherence.

Double medication	Therapeutic duplication is defined as the use of two or more drugs in the same ATC classification and with similar pharmacodynamic properties, which can lead to adverse drug events.

#### Pharmaceutical care plan

The purpose of the pharmaceutical care plan is to organize all of the pharmaceutical care agreed upon by the pharmacist, the GP, the practice nurse and the patient to achieve the goals of pharmacotherapy by addressing, resolving and preventing drug therapy problems. Together they formulate a care plan and the pharmacist specifically is responsible for the monitoring and the follow-up evaluation of the care plan and pharmacotherapy.

After establishing the drug therapy problems they are categorized based on a medical condition and on the drug therapy. When multiple drug therapy problems are present, they are prioritized based on the patient's perspective, focussing on those that causes the most concern and which one the patient is willing to address. The next step is to set the goals of the drug therapy and to agree on the interventions to achieve these goals. The interventions include start, stop or switch drug therapy, adjusting the dosage regimens or formulation, monitoring of drug therapy, referral to other clinician and individualized patient counselling and education. All these interventions are documented in the pharmaceutical care plan with detailed information on who is responsible for a certain action (GP, pharmacist or practice nurse), when the action will be taken and when it will be evaluated.

#### Monitoring and follow-up evaluation of care plan and pharmacotherapy

The focus of the monitoring and follow-up evaluation of pharmacotherapy is on the implementation of the pharmaceutical care plan. The pharmacist, the GP and if needed the patient, verify if all planned interventions are completed and they examine the outcomes of the documented drug therapy problems. They also assess if the goals of drug therapy are achieved. At this evaluation moment it is possible to plan new interventions to achieve previously documented goals of therapy or to solve previously identified drug therapy problems. The pharmacist documents the outcomes and possible new interventions in the care plan.

#### Control group

Patients in the control group receive usual care from their GP, pharmacist, practice nurse and other primary health care staff. This care consists of repeat prescriptions and medication surveillance according to the current clinical guidelines.

### Outcomes

The primary outcome of the study is the frequency of hospital admissions related to medication within the study period of 12 months of each patient. Two independent clinical pharmacists assess all admissions for a causal relationship between the reason for admission and the medication use, prior to the admission. This is done by reviewing the, blinded, discharge letter combined with the medical and medication data from an electronic Case Report Form (CRF), according to the adjusted Kramer algorithm[17].

The secondary outcomes are survival, quality of life, adverse drug events and severe adverse drug events. The quality of life is measured by the EuroQol EQ5D combined with the VAS (visual analogue scale) questionnaire at the start of the inclusion period and at the end of the inclusion period, after 12 months[18]. The pharmacist and the GP ask the patient at the end of the study period about symptoms of the past 3 months. They report and assess the adverse drug events according to the adjusted Kramer algorithm[17] combined with National Coordinating Council for Medication Error Reporting and Prevention (NCC MERP) scheme for categorisation of the severity of the events[19].

Other outcomes are the frequency and type of drug therapy problems (table 2) as documented in the pharmaceutical care plans over the intervention period. Two independent pharmacists code from the care plans, all the drug therapy problems, the proposed and executed intervention combined with the drug therapy. They meet to reach consensus when needed.

### Sample size

We expect to achieve an effect of 50% reduction of medication-related hospital admissions[2,20]. It seems possible to include 50 intervention patients from one intervention GP and 50 control patients from one control GP in one pharmacy practice, in a period of 12 months with a follow-up period of 12 months. To show a statistically significant difference between the intervention and the control arm with an expected prevalence of 0.01 (1%), we plan to include 14200 patients, 7100 in each arm, from at least 142 pharmacy practices and at least 284 GP practices to participate in the PHARM-study[21,22]. This is based on an alpha of 0.05 and a power (1-beta) of 0.8. It should be noted that the prevalence of HARM is expected to be higher in the group eligible for inclusion in the study.

#### Distribution of inclusion criteria

To determine the size of the population at risk and therefore eligible for inclusion, we searched the 'Kring-kubus-database'[23]. This database contains anonymous drug dispensing data from 100 (5.5%) community pharmacies, spread throughout the Netherlands, belonging to the franchise organization 'Kring-apotheek' (n = 330). In the Dutch community pharmacies, the drug history of individual patients can be considered as nearly complete because most patients visit only one single pharmacy[24]. This database includes drug-dispensing data from 719,022 patients with patient related information (age, sex, unique anonymous identifier), information on the dispensed drug (chemical substance coded according to the Anatomical Therapeutic Chemical (ATC) system[25] and dispensed product), date of dispensing, amount dispensed and prescribed dosage regimen. Patients who complied with the defined risk factors and therefore with the inclusion criteria as well (table 1), were selected from the database by using Microsoft Access. The use of the drug-dispensing data was performed in compliance with the Dutch privacy regulations.

**Figure 2:**
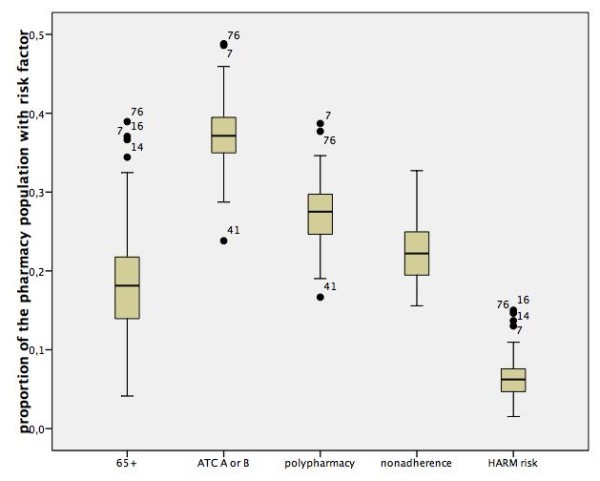
**Proportion of the patients at HARM risk in a pharmacy with the distribution of the risk factors in the pharmacies in the 'Kring-kubus' database**.

**Figure 3:**
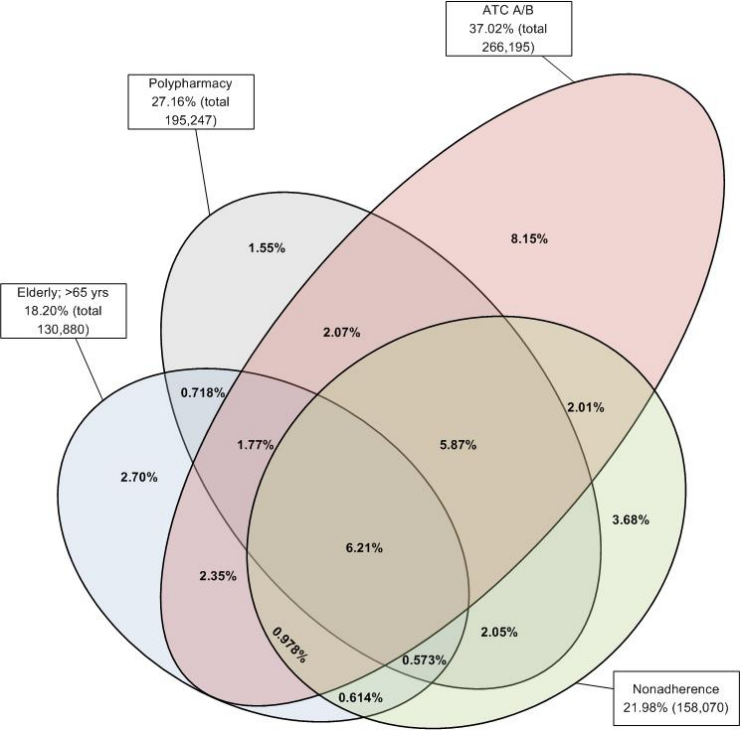
**The number of high-risk patients per risk factor and the overlap between the risk factors (total patient population: 719,022)**.

We found that in an average pharmacy practice 6.4% (range: 1.5 - 15.0%) of the patients had a high risk of being admitted to hospital with a medication-related cause, according to the HARM-risk model. In an average Dutch pharmacy practice the size of this high-risk population was around 450 patients, enough eligible patients for participating in the PHARM-study. The type of drug (ATC class A; alimentary tract and metabolism or ATC class B; blood and blood forming organs) risk factor was the most common risk factor in the population of a pharmacy: 37.2% (range: 23.8 - 48.8%), while non-adherence was the smallest risk factor in the population of a pharmacy: 22.4% (range: 15.6 - 37.7%). The relative numbers of high risk patients per risk factor in the Dutch pharmacies and the distribution of these risk factors are presented in the box plots in figure 2. The numbers of high-risk patients per risk factor and the overlap between the risk factors are presented in the Venn diagram in figure 3.

### Randomisation

Cluster randomisation takes place after informed consent of the participating GPs and pharmacists on a GP level and before the selection of patients. For every pharmacist a random selection of the intervention GP is made. The other participating GP becomes the GP who will include control patients.

### Data analysis

From each included patient data are collected in an electronic CRF containing: patient data, medical history and complaints, clinical data, quality of life and visual analogue scale, drug history with drug-allergies and drug-intolerabilities and a part with information on hospital admissions and adverse drug events. Detailed information on the data collected in the CRF can be found in table 5. The pharmacist and the GP complete the CRF with data from the pharmacy database, the GP database and from the interview with the patient. The collected data are based on literature and present guidelines[26-28].

**Table 5:** Data collection; content of case report form (CRF)

Part	Data content
Patient data	• patient ID* • age*^§^ • gender*^§^ • pharmacist*^§^ • GP*^§^ • weight^#/+^ • lengths^#/+^ • diet^+^ • alcohol consumption^+^ • smoking habits^+^ • cognition^$^ • abilities/disabilities^+^ • daily activities^+^ • former education\language^+^ • religion^+^ • home situation^+^ • home help^+^ • carer^+^ • medication aids^+^ • other relevant information (free text) ^+^

Medical data	• acute medical conditions (menu, coded with ICPC-code[31])^#^ • chronic medical conditions (menu, coded with ICPC-code[31])* ^#^ • current complaints or symptoms (menu, coded with ICPC-code[31]) ^#^ • significant past medical conditions or procedures^#^ • other relevant health information (free text) ^#^

Clinical data	• laboratory values^#^ • blood pressure^#^ • temperature^#^ • weight^#^ • other relevant measured clinical data^#^ available clinical data is collected from 12 months before inclusion till the end of the inclusion period.^#^

Medication data	full drug history: all dispensed drugs from 12 months before inclusion till the end of the inclusion period^§^, containing information on: • chemical substance (menu, coded with ATC-code[25]) ^§^ • dispensed product (extracted from pharmacy database)* ^§^ • dispensed amount (extracted from pharmacy database)* ^§^ • dispensing date (extracted from pharmacy database)* ^§^ • prescribed dose (extracted from pharmacy database)* ^§^ • over the counter (OTC) drugs^§, +^ • vitamins, dietary or nutritional supplements^§, +^ • other health products^§, +^ • medical devices^§, +^ • drug allergies^§, +^ • intolerabilities or adverse drug events^§, +^

Study outcomes	• hospital admissions (discharge letter as appendix)^#^ • date of each admissions^#^ • reason for eacht admission^#^ • quality of life; EQ5D score (at t = 0* and t = 12 months)^+^ • visual analogue scale score(at t = 0* and t = 12 months) ^+^ • adverse drug events (ADE) with symptom, related drug and timing information (at t = 12 months) ^+, #^ • severity of the ADE^+, #^

Pharmaceutical care plan^$^	• DTPs (according to the Subjective-Objective-Assessment-Plan (SOAP) notes[29]) • therapy goals • priorities • interventions • clinician who is executing the action • date of action • date of evaluation of the action

* obligatory; ^$ ^for intervention patients only

^# ^Obtained from medical record

^§ ^Obtained from medication record

^+ ^Obtained from the patient during pharmaceutical anamnesis or questionnaire

For the intervention patients the CRF also contains a separate part for documenting the pharmacotherapy review, the pharmaceutical care plan and monitoring and follow up evaluation of pharmacotherapy. In this plan information is organised according to drug therapy related problems with the Subjective-Objective-Assessment-Plan (SOAP) notes method[29]. The assessment and evaluation of the different drug therapy problems are subsequently documented and prioritised and the subsequent interventions are documented with specific information on who of the clinicians is taking the action, when the action is taken and when the action is evaluated.

Data from the digital Excel CRF and from a Microsoft Access database are combined and further analysed by using R, version 2.6.2. In R mixed-effects Cox models[22] are designed to study the effect of the intervention on hospital admissions related to medication and the effect on survival, quality of life and adverse drug events. All available patient data are included. The pharmacist and the GP are integrated as random effects in the models. P-values < 0.05 are regarded as statistically significant. The two-sided 95% bootstrap percentile confidence intervals are computed using 1000 replications. Bootstrap samples are obtained by random sampling GP's or patients with replacement from the population. To assess the model's goodness of fit we create plots of outcome versus follow-up time, versus number of diseases and examined residuals. The influence of the baseline characteristics such as age, gender, number of physicians, number of diseases, number of drug prescribed, refill rate and follow-up time are also analysed using linear mixed-effects models or generalized linear mixed-effects models.

## Discussion

The PHARM-study looks into the effect of the complex pharmaceutical care process on medication related hospital admissions. This patient centred pharmaceutical care process is highly patient individualized, includes several steps (figure 1) and is conducted in a primary care setting. This care process can only be partly structured in a study protocol because it is dependent on patients' autonomy, the performance of the clinicians and on the cooperation between them. These issues cannot be completely restrained to a protocol and will be a reflection of real clinical practice. In the PHARM-study the effectiveness of the pharmaceutical care process will be studied in daily clinical practice and due to this the study is a pragmatic trial. Such a trial is suitable to evaluate effectiveness rather than to measure efficacy[30].

A key methodological issue in pragmatic trials is balancing internal and external validity[30]. This issue is addressed for the PHARM-study in the study design and evaluation protocol. External validity, or generalizability, is achieved by allowing the GPs and the pharmacists to implement the intervention in their own manner within their daily practice. External validity is also addressed by having very few exclusion criteria and general inclusion criteria. The included high-risk patients are a heterogeneous group, with multiple and varying morbidities and medication usage. Internal validity is maintained by selecting intervention patients from another general practitioner than the control patients, to reduce contamination bias by ensuring that GPs do not apply strategies and knowledge used in the intervention patients to control patients.

We expect this study to have several other strengths besides internal and external validity. First, we have gained experience during several previous pilot projects and because of this we were able to optimize the intervention by providing a structure for the pharmacotherapy review. We have also optimized the CRF for documentation of the pharmaceutical care and the planned intervention in drug therapy to guarantee the continuity of care. Since pharmacotherapy and medical services become more complex, creating a comprehensive documentation is required to facilitate collaboration between members of the health care team and ensure continuity of care. Second, the pharmacist and the GP are offered a training regarding the study protocol, the documentation and how to perform a structured pharmacotherapy review. A third strength is the large group of patients, which makes it possible to measure a possible effect on medication related hospitalisations. A fourth strength is the extensive intervention during a period of 12 months in an interdisciplinary setting in cooperation between the patient and the own GP and pharmacist, which makes it more likely to solve all major drug therapy problems.

This study also has some limitations. First, selection bias may occur by just including patients who are willing to cooperate in the intervention. However, the same selection will take place in the control group because they also have to cooperate in completing the questionnaires. Second the selection of the participating pharmacist and GPs is based on voluntary participation. Only motivated couples of GPs and a pharmacist who established a good cooperation will participate in the study, which hampers the generalizability. But in case the intervention shows an effect, these participating GPs and pharmacists can be a role model for their colleagues in the region. A third limitation is the extensive and therefore demanding intervention. It is thinkable that it will not be implemented as described in the protocol because this is not possible or feasible in daily practice. A fourth limitation is the selection of intervention and control patients on a general practice level but from the same pharmacy which implies that control patients are cared for by the same pharmacist as the intervention patient. This possibly leads to contamination bias, but we do not expect that the pharmacist will provide other care than usual care to the control patients. A fifth limitation is the assessment of the ADEs which will be done by the patient's own pharmacist and GP who are not blinded to the assignment to intervention or control group. Knowing to which group the patient is assigned, may possibly affect their judgement of ADEs, which can potentially lead to the detection of more ADEs in the control group. On the other hand an advantage may be that they know their patients so they can make a better judgement if the symptoms are related to the medication or to a disease.

The PHARM-study is one of the largest controlled trials to study the effect of the total pharmaceutical care process, including a pharmaceutical anamnesis, followed by a pharmacotherapy review, a pharmaceutical care plan, monitoring and follow up evaluation of pharmacotherapy, in an ordinary integrated primary care setting on medication related hospital admissions. Few controlled trials in this area have been performed and none have investigated the extensive intervention of the total pharmaceutical care process, performed in cooperation of the patient, the pharmacist, the GP and the practice nurse, in this group of high risk patients, at the scale of this study either in terms of the number of practices or the number of patients. The PHARM-study should provide evidence as to whether such a pharmaceutical care process should be implemented in a primary care setting in the Netherlands.

## Competing interests

The authors declare that they have no competing interests.

## Authors' contributions

All authors are responsible for interpretation of the data and are involved with drafting and critically reviewing the manuscript. AL, FK, AG, AJ, AG, TE and PB are responsible for study design and study implementation. AL and SB (see acknowledgements) are responsible for analysis of the population at risk. All authors have read and approved the final manuscript.

## Pre-publication history

The pre-publication history for this paper can be accessed here:

http://www.biomedcentral.com/1472-6963/11/4/prepub
